# MicroRNAs with non-additive expression in the ovary of hybrid hens target genes enriched in key reproductive pathways that may influence heterosis for egg laying traits

**DOI:** 10.3389/fgene.2022.974619

**Published:** 2022-09-30

**Authors:** Adamu Mani Isa, Yanyan Sun, Yunlei Li, Yuanmei Wang, Aixin Ni, Jingwei Yuan, Hui Ma, Lei Shi, Hailai Hagos Tesfay, Jing Fan, Panlin Wang, Jilan Chen

**Affiliations:** ^1^ Key Laboratory of Animal (Poultry) Genetics Breeding and Reproduction, Ministry of Agricultural and Rural Affairs, Institute of Animal Science, Chinese Academy of Agricultural Sciences, Beijing, China; ^2^ Department of Animal Science, Usmanu Danfodiyo University, Sokoto, Nigeria

**Keywords:** crossbreeding, heterosis, egg (production), clutch size, miRNA, dominance

## Abstract

Heterosis has been extensively exploited in chicken breeding to improve laying traits in commercial hybrid stock. However, the molecular mechanisms underlying it remains elusive. This study characterizes the miRNAome in the pre-hierarchical follicles of purebred and hybrid laying hens, and investigate the functions of miRNAs with non-additive expression in the pre-hierarchical follicles as they modulate heterosis for egg number and clutch size. To achieve that aim, White Leghorn and Rhode Island Red chicken lines were reciprocally crossed to generate hybrids. The crossbreds demonstrated heterosis for egg number and clutch size, and pre-hierarchical follicles from 4 birds of each genotype were collected at 53 weeks of age. Mode of miRNA expression was characterized after miRNA sequencing. A total of 50 miRNAs including 30 novel ones, were found to exhibit non-additive expression. Dominance was the predominant mode of expression exhibited by majority of the miRNAs. Functional analysis of target genes of the known miRNAs with non-additive expression revealed Gene Ontology terms related to regulation of transcription, metabolic processes and gene expression. KEGG and REACTOME pathways including hedgehog, cellular senescence, wnt, TGF-β, progesterone-mediated oocyte maturation, oocyte meiosis, GnRH signaling, signal transduction and generic transcription, which can be linked to primordial follicle activation, growth and ovulation, were significantly enriched by target genes of miRNAs with non-additive expression. Majority of the genes enriched in these biological pathways were targeted by gga-miR-19a, gga-miR-19b, gga-miR-375, gga-miR-135a, and gga-miR-7 and 7b, thus, revealing their synergistic roles in enhancing processes that could influence heterosis for egg number and clutch size in hybrid hens.

## Introduction

Heterosis remains a central theme in the field of poultry breeding, and previous investigations established non-additive gene action as the primary cause. In chickens, heterosis is accomplished through crossbreeding genetically distinct lines and breeds such that the average performance of the crossbred population is superior to the mid performance of the purebred parental lines expected under additive gene assumptions. In addition to heterosis, breed complementarity is also exploited by crossbreeding. The success of crossbreeding schemes largely depends on crossing of genetically diverse lines (Amuzu-Aweh, 2020).

Egg laying efficiency typified by high laying rates, large number of eggs and larger clutches seem to be the traits that are enhanced in the hybrids laying birds, and were linked to well-orchestrated and organized follicular hierarchy in the hens (Johnson, 2012). The selection process involves recruitment from pre-hierarchical pool, follicles into the pre-ovulatory hierarchy. The changes that occur after follicle recruitment during the pre-ovulatory period were propound and differ even between pre-ovulatory follicles. The dynamics in gene expression profiles of pre-ovulatory follicles with its attendant variation between pullets of the same genotype poses great challenge to transcriptomics studies. Prior to selection event, many pre-hierarchical follicles either undergo atresia or remain steroidogenic incompetent in an effort to preserve the sanctity of small viable cohorts of pre-selected follicles associated with follicular reserve and subsequently, clutches of eggs (Johnson, 2015).

Previous studies have elucidated the mechanisms of maintaining the state of primordial follicles by local factors and intracellular pathways. This occurs via the action of multiple activators including growth differentiation factor 9 (GDF9), anti-mullerian hormone (AMH), zona pellucida 2 (ZP2), wingless-type MMTV integration site family member 4 (WNT 4) (Zhang et al., 2019), and recently, PPAR pathway (Yoon et al., 2020).

The discovery of the first microRNA (miRNA) gene member (lin4) in the early 90s (Lee et al., 1993) has ushered an ever-expanding field of miRNA research with new members being reported every day. MicroRNAs are a class of small non-coding RNAs (∼22 nt) that regulate gene expression at a post-transcriptional level through complementary base pairing with the target mRNA, leading to degradation of mRNA and eventually repressing its translation (Riffo-Campos et al., 2016). Many investigations have shown that miRNAs are involved in the regulation of various pathways and exert influence on a wide variety of phenotypes. To date, there are 1,235 chicken miRNAs cataloged in the miRDB database (http://mirdb.org/statistics.html) and the number will continue to increase with the decline in the cost of high throughput sequencing, commensurate with Moore’s law (Brock and Moore, 2006) and increasing curiosity of molecular scientist to address myriads of complex molecular phenomena. The array of functions miRNAs perform in the translation of coded information in the DNA to phenotype lies with its unique ability to bind to the canonical site of other RNA species called microRNA response elements (MRE), which allows it to interact with a wide variety of RNA species (including mRNAs, lncRNAs, and circRNAs). MiRNAs are therefore central molecules in the theory of cross-talking of ceRNAs proposed at the beginning of the last decade (Salmena et al., 2011). This class of RNAs represses the expression of mRNAs thereby lowering the expression of the genes encoding the larger species of ceRNAs.

In chickens, the roles of miRNAs in the expression of phenotypes including sperm motility in roosters (Liu et al., 2018) and egg number in laying hens (Zhang et al., 2017) have been documented in recent times. Despite the growing body of literature in the field of miRNA biogenesis, identification and target prediction on one hand and intensive search for molecular mechanisms of heterosis in domestic chickens (Mai et al., 2019; Zhuo et al., 2019) on the other hand, there is still a gap in literature on the mode of inheritance and possible influence of miRNAs on heterosis for egg number and clutch size in laying chickens. This study therefore, aims to identify miRNAs with non-additive mode of expression which may influence heterosis for egg number and clutch size in laying hens.

## Results

### Phenotypic data

**FIGURE 1:**
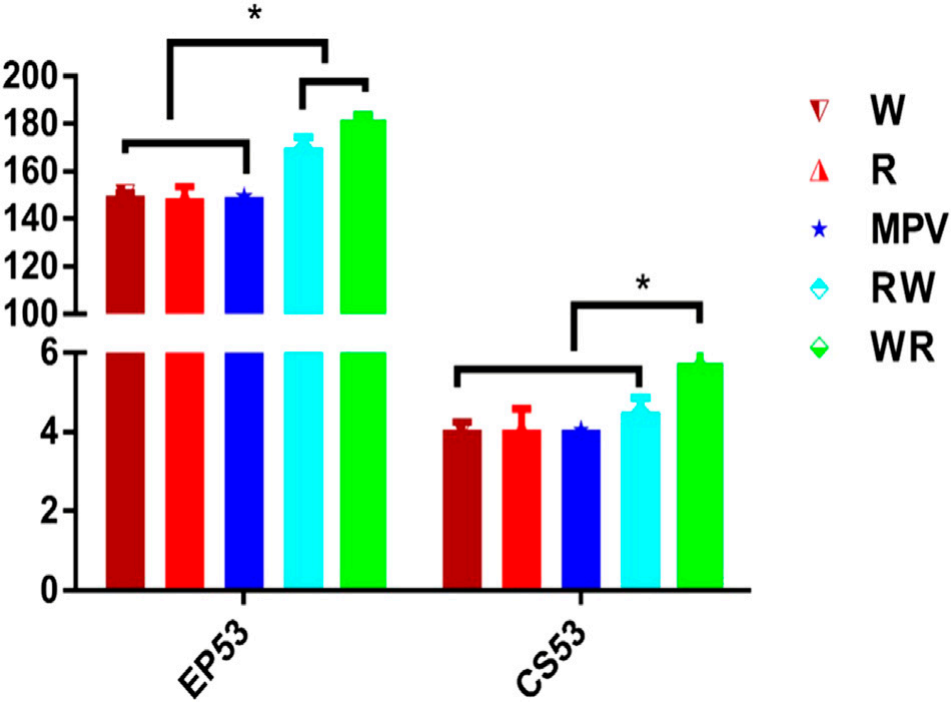
Egg number and clutch size at 53 weeks of age for parental lines and their reciprocal hybrids. Mean values with an asterisk (*) on top of them were significantly different (Tukey-Kramer HSD *p* ≤ 0.05). W: Purebred White Leghorn, R: Purebred Rhode Island Red, RW: Rhode Island Red × White Leghorn cross, WR: White Leghorn × Rhode Island Red cross, MPV: Mid-parent value.

Number of eggs laid by the White Leghorn (W) and Rhode Island Red (R) parental purebred lines were significantly less than (*p* < 0.001) the number laid by their reciprocal hybrids at 53 weeks of age (Figure 1). The synthesized mid-parent value (MPV) for the purebred was similar to the two parental lines. Heterosis for egg number was 14.1 and 18.5% in Rhode Island Red × White Leghorn (RW) and White Leghorn × Rhode Island Red (WR) respectively. Furthermore, heterosis for clutch size was 12.29 and 38.43% in RW and WR hybrids respectively.

### Overview of miRNAome mapping statistics

MicroRNA sequences generated from the 16 libraries constructed from RNA extracted from pre-hierarchical white follicles in the two purebred (R and W) and their reciprocal hybrids (RW and WR) yield a total of 291, 129, 265 reads after filtering for quality (Phred score >20), size selection (18–30 nt) and trimming of adapter sequences. Out of this, 272,842, 977 (93.66%) reads aligned to the chicken genome built GRCg6a of the Ensembl database and were preserved for further analysis. Specifically, 169, 706, 629 reads (62.30%) mapped with a perfect match while the remaining 103, 136, 348 reads (37.8%) mapped with a single nucleotide mismatch. For each library, more than 90% of the clean reads were successfully mapped to the chicken genome (Supplementary Figure S1).

Furthermore, mapped reads belonging to other species of non-coding RNAs including rRNA (8,103,438 (2.97%)), tRNA (4,251,546 (1.55%)), snRNA (176,885 (0.06%)), snoRNA (5,646,100 (2.07%)) and others (563,900, (0.21%)) collectively accounted for 6.44% of the total reads, and were discarded (Supplementary Table S1). Similarly, reads that mapped to the low diversity region of the genome including SINES, LINES, and LTR which collectively accounted for 0.83% of the total raw reads were not retained in the subsequent analyses.

**FIGURE 2:**
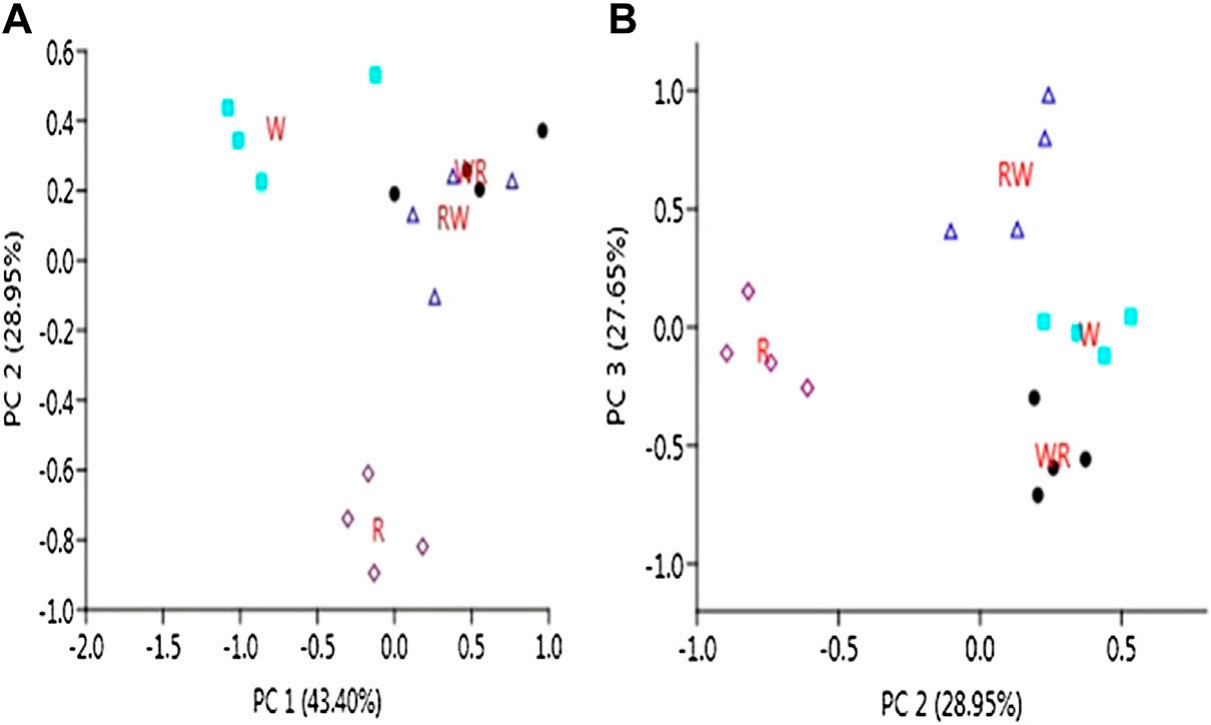
Separation of the pure lines and reciprocal hybrids by PCA **(A)**: PC1 vs. PC2 plot **(B)**: PC2 vs. PC3 plot.

A total of 899 and 289 known and predicted novel miRNAs were found to be expressed in the pre-hierarchical follicles. After filtering out the lowly expressed miRNAs with <10 read counts per million in all the libraries, a total of 595 known and 235 novel miRNAs were preserved for further downstream analyses (Supplementary Table S2). Principal component analysis (PCA) of the retained clean reads depicts clear separation of the four genetic groups (Figure 2). PC1 *vs.* PC2 plot assigned the genotypes into three clusters; two clusters for W and R purebred parental lines and another cluster for the two reciprocal hybrids (RW and WR). Furthermore, PC2 *vs.* PC3 plot separates the genotypes into four distinct clusters.

MiRNAs with abundant expression (∼1 million raw reads/per library) in the ovary of laying hens at 53 weeks of age include gga-miR-143-3p, gga-miR-148a-3p, gga-miR-26a-2-5p, gga-miR-26a-5p, gga-miR-99a-5p, gga-miR-21-5p and gga-miR-10a-5p. The most abundant miRNA (gga-miR-143-3p) had raw reads between 9 and 13 million reads, accounting for 3.9, 3.8, 4.9 and 4.3% of all expressed miRNAs in R, W, RW and WR genotypes respectively. Twenty most abundantly expressed known miRNAs expressed in the pre-hierarchal follicles in the purebred and hybrids laying chickens were same and are presented in Table 1.

**TABLE 1:** The 20 most abundantly expressed miRNAs in the ovary of purebred and hybrid chickens.

miRNA	R		RW		W		WR	
	Read counts	% of Total	Read counts	% of Total	Read counts	% of Total	Read counts	% of Total
gga-miR-143-3p	10688755	3.92	13462849	4.93	9219675	3.38	11713640	4.29
gga-miR-148a-3p	8918917	3.27	9317996	3.42	5912664	2.17	10132356	3.71
gga-miR-26a-2-5p	4442976	1.63	4900246	1.80	4018858	1.47	6210366	2.28
gga-miR-26a-5p	4442972	1.63	4900244	1.80	4018853	1.47	6210361	2.28
gga-miR-99a-5p	3900383	1.43	5325620	1.95	3461332	1.27	6754169	2.48
gga-miR-21-5p	4783473	1.75	3356219	1.23	2331764	0.85	3730281	1.37
gga-miR-10a-5p	2451540	0.90	2223960	0.82	2780188	1.02	2534866	0.93
gga-miR-146c-5p	1266042	0.46	1289721	0.47	1272549	0.47	1151559	0.42
gga-miR-145-5p	1130629	0.41	935879	0.34	1875808	0.69	1036686	0.38
gga-miR-199-3p	1107227	0.41	1352070	0.50	841458	0.31	1515098	0.56
gga-miR-126-3p	1111832	0.41	1235579	0.45	856975	0.31	1399615	0.51
gga-miR-100-5p	969498	0.36	1193374	0.44	801364	0.29	1205579	0.44
gga-miR-146b-5p	1278331	0.47	856633	0.31	1207839	0.44	821916	0.30
gga-let-7a-5p	999090	0.37	1048792	0.38	926779	0.34	1176787	0.43
gga-let-7j-5p	999090	0.37	1048792	0.38	926779	0.34	1176787	0.43
gga-let-7g-5p	918332	0.34	956672	0.35	919716	0.34	1035672	0.38
gga-miR-101-3p	774947	0.28	1272755	0.47	750151	0.27	976429	0.36
gga-let-7f-5p	940817	0.34	906388	0.33	918234	0.34	984032	0.36
gga-miR-125b-5p	637100	0.23	851730	0.31	605576	0.22	1125438	0.41
gga-let-7i	699908	0.26	649135	0.24	725435	0.27	666154	0.24

### Differentially expressed miRNAs and their mode of inheritance

**FIGURE 3:**
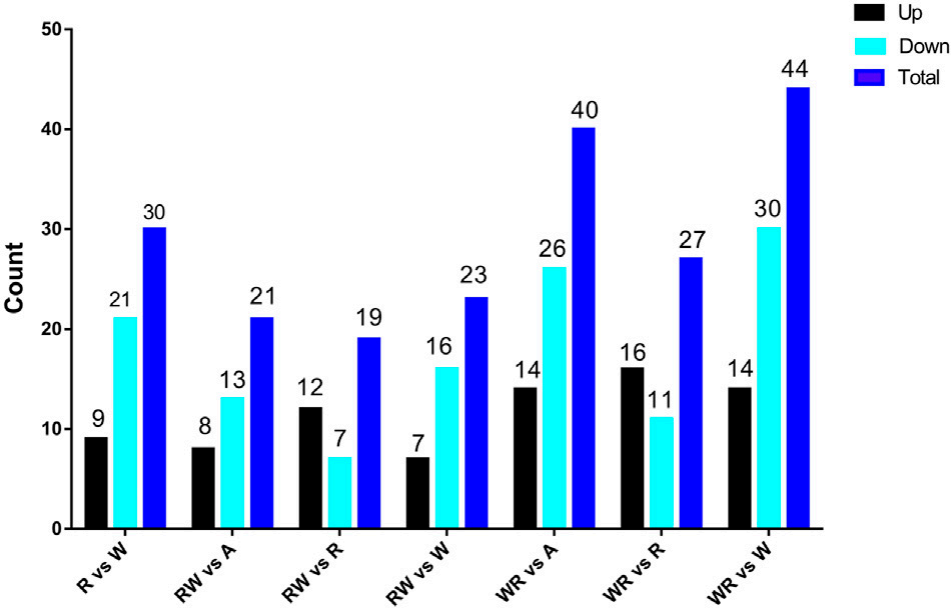
Differentially expressed miRNAs among parental lines (W and R), hybrids (WR and RW) and synthesized average expression of the parents (A).

Pair-wise comparisons of miRNA expression between the pre-hierarchical follicles in the ovaries of the reciprocal hybrids and their parental purebreds on one hand and between the hybrids and synthesized mid-parent average on the other hand are presented in Figure 3.

Comparing miRNA expression in the two parental purebred lines, 30 differentially expressed miRNAs (DEMiRs) were detected, including nine upregulated and 21 downregulated miRNAs. In RW vs. A, 21 DEMiRs were identified, 8 and 13 were upregulated and downregulated respectively. In RW vs R, a total of 19 DEMiRs were identified while 23 DEMiRs were detected in RW vs W. Further, WR vs. R yielded 65 DEMiRs consisting of eight upregulated and 57 downregulated miRNAs, and 139 miRNAs showed differential expression in WR vs. R. In WR vs. W, 127 DEMiRs were detected including 54 upregulated and 73 downregulated miRNAs.

**FIGURE 4:**
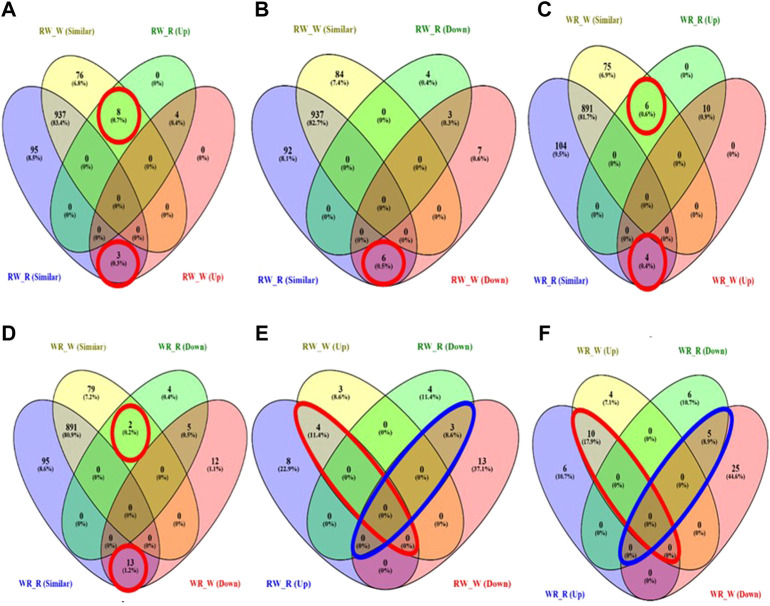
Venn diagram showing DEMiRs according to their pattern of inheritance **(A)** High parent dominance in RW **(B)** Low-parent dominance in RW **(C)** High parent dominance in WR **(D)** Low-parent dominance in WR **(E)** Overdominance (enclosed in red) and underdominance (enclosed in blue) expression of miRNAs in RW **(F)** Overdominance (enclosed in red) and underdominance (enclosed in blue) expression of miRNAs in WR.

Overall, all pairwise comparisons yielded 70 unique DEMiRs. Further classification of the DEMiRs based on their mode of inheritance pattern was achieved by overlapping the DEMiRs in Venn diagrams. Total of 20 DEMiRs exhibited additive mode of expression (Supplementary Figure S2), while the remaining unique DEMiRs exhibited non-additive mode of expression (Figure 4). Of these, 20 known miRNAs were grouped in to 14 miRNA family clusters (Table 2), and dominance mode of expression pattern was exhibited by 19 of them. The remaining 30 were suggested novel miRNAs (Supplementary Table S4). Overall, 14, 17, 13 and six miRNAs exhibited high-parent dominance, low parent dominance, over-dominance and under-dominance inheritance patterns respectively (Table 2; Supplementary Table S3).

**TABLE 2:** Known miRNAs abundance and their non-additive modes of expression in the ovary of purebred White Leghorn, Rhode Island Red, and their reciprocal hybrids.

miRNA	Raw read counts				Mode of expression
	R	W	RW	WR	
gga-miR-10b-5p	56014	232040	49575	60899	LP Dominance both
gga-miR-122-5p	49067	8141	11986	6515	LP Dominance in WR
gga-miR-135a-5p	2515	4911	2724	1946	LP Dominance in WR
gga-miR-145-5p	1130629	1875808	935879	1036686	LP Dominance in RW
gga-miR-1684a-3p	6	437	280	316	HP Dominance in both
gga-miR-1684b-3p	869	21	571	409	HP Dominance in both
gga-miR-1720-5p	28	39	61	265	HP Dominance in WR
gga-miR-1747-5p	229	1	81	107	HP Dominance in both
gga-miR-19a-3p	28429	64340	28105	27508	LP Dominance in both
gga-miR-19b-3p	32190	72366	34010	32786	LP Dominance in WR
gga-miR-205a	1448	3705	2320	1009	LP Dominance in WR
gga-miR-217-5p	40	606	110	94	LP Dominance in RW
gga-miR-34b-3p	430	1731	262	154	LP Dominance in both
gga-miR-34b-5p	1892	5420	960	535	LP Dominance in WR
gga-miR-34c-3p	133	261	86	53	LP Dominance in WR
gga-miR-34c-5p	1889	5414	960	535	LP Dominance in WR
gga-miR-365-3p	744	1522	729	636	LP Dominance in both
gga-miR-375	589	610	4698	60891	Over-dominance in WR
gga-miR-7	61657	99612	63096	272947	HP Dominance in WR
gga-miR-7b	105796	149761	67781	72170	LP Dominance in both

LP, dominance: low parent dominance; HP, dominance: high parent dominance.

### Functional enrichment analysis of the target genes of the miRNA with a non-additive mode of expression

**FIGURE 5:**
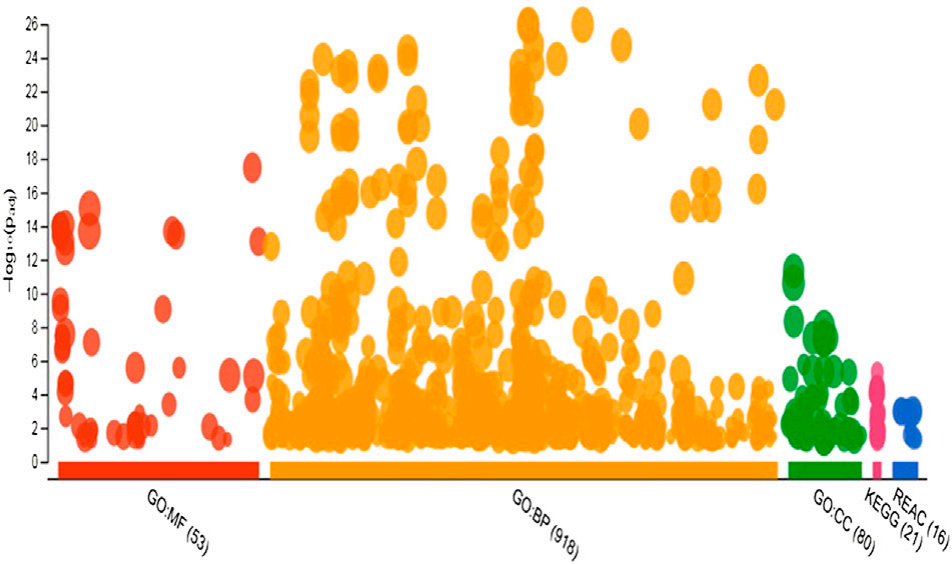
Manhattan plot showing number of significantly enriched terms in GO, KEGG and REACT databases for target genes of miRNAs with non-additive expression.

A total of 970 unique genes were identified as potential targets for 11 known miRNA with non-additive expression (Supplementary Table S4). Target genes with aggregate PTC score ≥50 were screened against the mRNA transcriptome of the pre-hierarchical follicles obtained by sequencing same sample from where miRNAome was sequenced. This yields a list of 970 unique mRNAs, which were uploaded into g. profiler for functional analysis including GO, KEGG and REAC. Overview of the result of gene enrichment analysis in GO, KEGG and REAC databases is depicted in Figure 5. The enriched GO terms consist of 918 biological processes (BP), 53 molecular functions (MF) and 80 cellular components (CC) (Supplementary Table S5). Specifically, GO annotation enrichment showed that target genes of miRNAs with non-additive expression were associated with regulation of cellular process (GO: BP term; FDR = 1.12 *×* 10^*–*26^), nucleus (GO:CC term; FDR = 4.77 *×* 10^*–*12^) and transcription regulatory activity (GO:MF term; FDR = 3.4 *×* 10^*–*18^).

**FIGURE 6:**
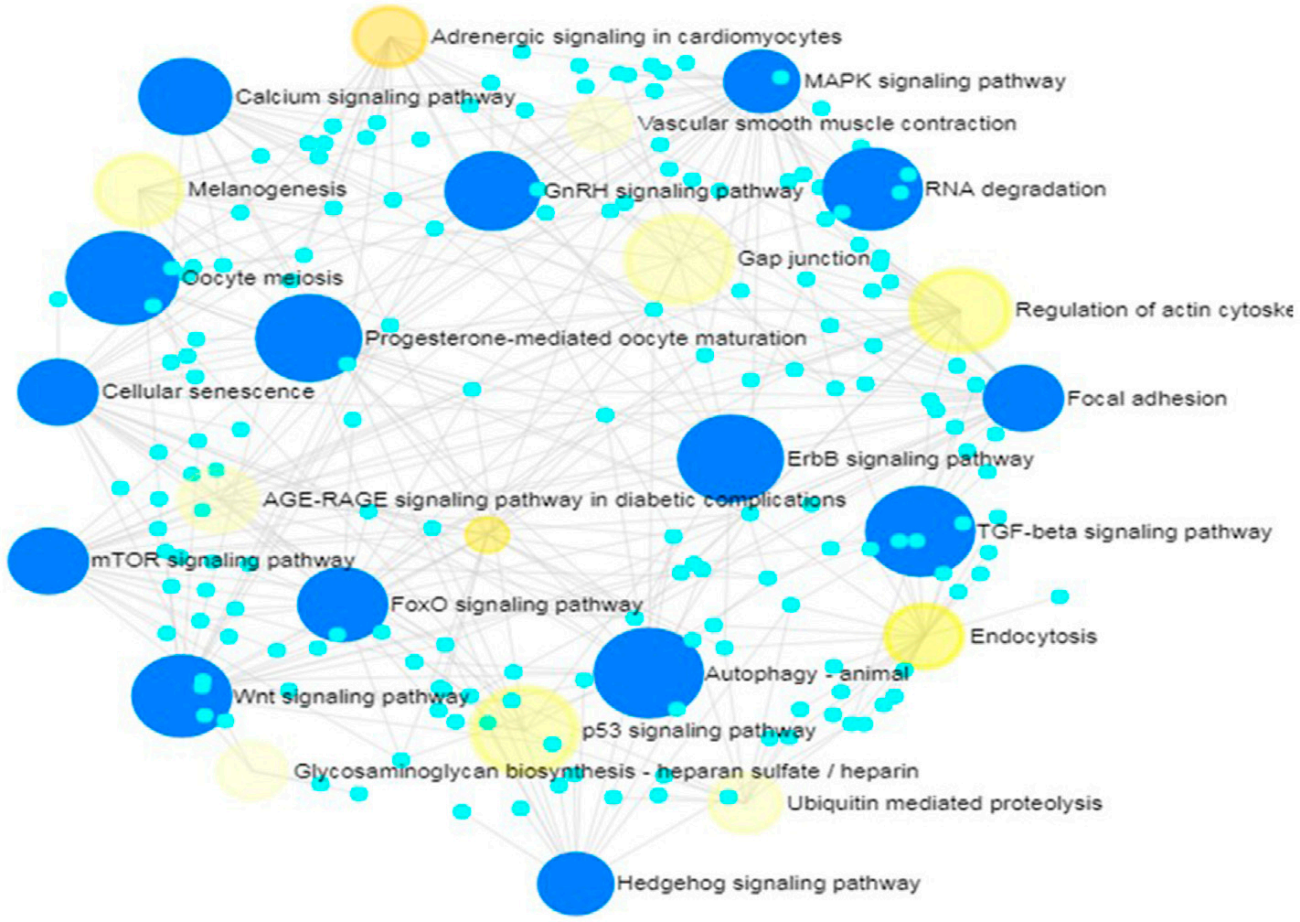
Network of enriched KEGG pathways for target genes of non-additively expressed miRNAs. Small cyan circles represent target genes of miRNAs with non-additive expression; Yellow circles represent KEGG pathways enriched by the target genes with *p*-values<0.05; Large blue circles represent KEGG pathways enriched by the target genes with corrected *p*-values<0.05.

Further, 21 and 16 pathways respectively in KEGG and REACTOME databases were significantly enriched by target genes of miRNAs with non-additive expression. In KEGG database, the pathways include Hedgehog signaling, cellular senescence, focal adhesion, FoxO signaling Wnt signaling, MAPK signaling, mTOR signaling, insulin signaling, TGF-β signaling, oocyte meiosis, autophagy, progesterone-mediated oocyte maturation and GnRH signaling (Table 3). These pathways share common genes and were interconnected (Figure 6).

**TABLE 3:** Pathways enriched in the KEGG database for target genes of miRNAs with non-additive mode of expression.

KEGG ID	KEGG pathway	P- Adjusted
KEGG:04340	Hedgehog signaling pathway	4.83 *×* 10^*–*6^
KEGG:04218	Cellular senescence	3.75 *×* 10^*–*5^
KEGG:04068	FoxO signaling pathway	3.75.59 *×* 10^*–*5^
KEGG:04310	Wnt signaling pathway	4.23 *×* 10^*–*5^
KEGG:04261	Adrenergic signaling in cardiomyocytes	4.23 *×* 10^*–*5^
KEGG:04010	MAPK signaling pathway	8.49 *×* 10^*–5*^
KEGG:04510	Focal adhesion	8.94 *×* 10^*–*5^
KEGG:04150	mTOR signaling pathway	1.22 *×* 10^*–*4^
KEGG:04910	Insulin signaling pathway	1.13 *×* 10^*–3*^
KEGG:04114	Oocyte meiosis	1.33 *×* 10^*–3*^
KEGG:04916	Melanogenesis	1.75 *×* 10^*–*3^
KEGG:04012	ErbB signaling pathway	332 *×* 10^*–*3^
KEGG:04140	Autophagy - animal	3.32 *×* 10^*–*3^
KEGG:04810	Regulation of actin cytoskeleton	1.40 *×* 10^*–*2^
KEGG:04371	Apelin signaling pathway	1.4136 *×* 10^*–*3^
KEGG:04350	TGF-β signaling pathway	2.56*×*10^*2*3^
KEGG:04144	Endocytosis	2.74 *×* 10^*–*2^
KEGG:04914	Progesterone-mediated oocyte maturation	2.74 *×* 10^*–*2^
KEGG:04330	Notch signaling pathway	3.34 *×* 10^*–*2^
KEGG:04912	GnRH signaling pathway	3.65 *×* 10^*–*2^
KEGG:04270	Vascular smooth muscle contraction	4.82 *×* 10^*–*2^

In the REACTOME database, enriched pathways include glucagon-like peptide (GLP1) regulates insulin secretion, post-transcriptional regulation by small RNA, generic transcription, gene expression, signal transduction, signal by WNT and regulation of insulin secretion (Table 4).

**TABLE 4:** REACTOME terms enriched by target genes of miRNAs with non-additive modes of expression in crossbred laying hens.

TERM ID	REACTOME term	P- Adjusted
REAC:R-GGA-381676	Glucagon-like Peptide-1 (GLP1) regulates insulin secretion	0.001040923
REAC:R-GGA-163685	Integration of energy metabolism	0.001040923
REAC:R-GGA-426496	Post-transcriptional silencing by small RNAs	0.001040923
REAC:R-GGA-162582	Signal Transduction	0.001040923
REAC:R-GGA-212436	Generic Transcription Pathway	0.001040923
REAC:R-GGA-9006934	Signaling by Receptor Tyrosine Kinases	0.001040923
REAC:R-GGA-422356	Regulation of insulin secretion	0.001040923
REAC:R-GGA-112316	Neuronal System	0.001040923
REAC:R-GGA-74160	Gene expression (Transcription)	0.001040923
REAC:R-GGA-73857	RNA Polymerase II Transcription	0.001892943
REAC:R-GGA-388844	Receptor-type tyrosine-protein phosphatases	0.013307074
REAC:R-GGA-392517	Rap1 signalling	0.013307074
REAC:R-GGA-6794362	Protein-protein interactions at synapses	0.025576206
REAC:R-GGA-195721	Signaling by WNT	0.037665791
REAC:R-GGA-8878159	Transcriptional regulation by RUNX3	0.047377253
REAC:R-GGA-5627117	RHO GTPases Activate ROCKs	0.047377253

### Regulatory network for miRNA with non-additive expression and their target genes

**FIGURE 7:**
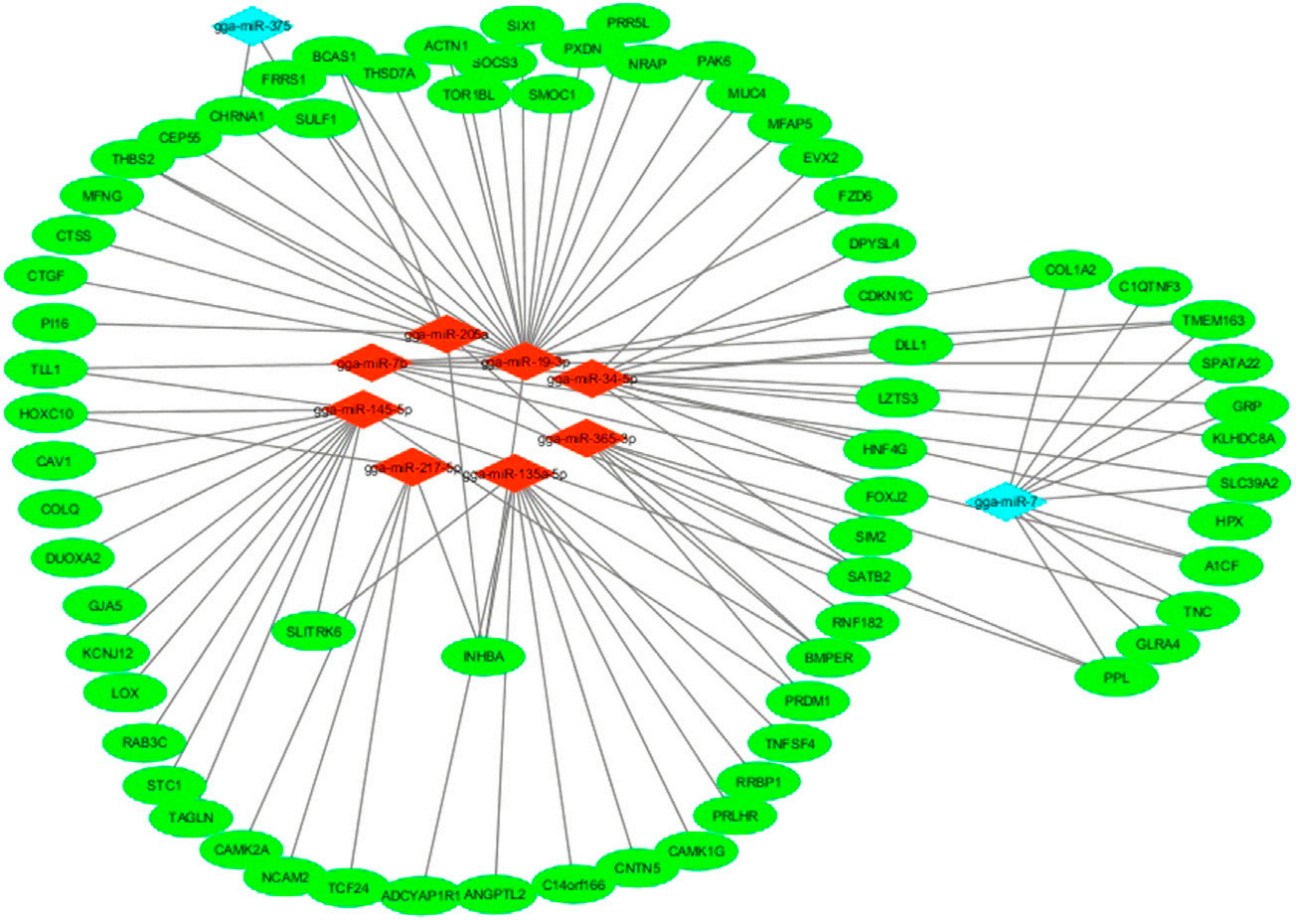
mRNA-miRNA regulatory network in the pre-hierarchical follicles of purebred and hybrid laying hens. Red diamonds represent miRNAs with low parent dominance expression; Cyan diamonds represent miRNAs with either high-parent dominance or over-dominance expression in the hybrids; Green ellipses represent target genes of miRNAs with non-additive expression.

The co-expression network for miRNAs with non-additive expression and their target genes is presented in Figure 7. The network revealed that more than one gene can be targeted by one miRNA. MicroRNAs that exhibited low-parent dominance such as gga-miR-19-3p targeted many genes in the follicles. Contrarily, gga-miR-375 with overdominance in WR genotype targeted only CHRNA1 and FRRS1 genes.

### Real-time quantitative PCR validation

**FIGURE 8:**
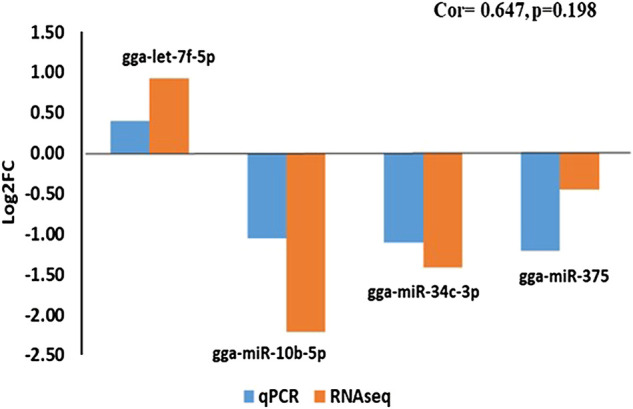
RT-qPCR validation of four miRNAs that exhibited non-additive modes of expression identified using Illumina small RNA deep sequencing.

Expression analysis of the four miRNAs revealed that pattern of expression was consistent between the Illumina small RNA sequencing and real-time quantitative PCR (RT-qPCR) with a correlation co-efficient of 0.647 (Figure 8).

## Discussion

Mode of miRNAs inheritance in reciprocal hybrids and their influence on heterosis for egg number and clutch size traits in laying chickens was not hitherto documented. By sequencing miRNAome of pre-hierarchical follicles of laying hens, we delineate the modes of miRNAs expression in parental purebred lines (R and W) and their reciprocal crossbreds (RW and WR). We identified 595 known miRNAs and successfully predicted 325 novel miRNAs that were expressed in the ovary of the hybrid laying hens and their purebred parental lines. MicroRNAs with highest abundance were gga-miR-143-3p and gga-miR-148a-3p, which together accounted for approximately 8% of the total expressed miRNAs in the pre-hierarchical follicles of the hens. The top five most abundantly expressed miRNAs constitute approximately 10–15% of the expressed miRNAs in the purebred and the hybrids respectively. Interestingly, all of them were known miRNAs, suggesting that known miRNAs were most abundantly expressed while predicted novel miRNAs were lowly expressed, and hence, the inability to detect them in previous investigations. This is consistent with previous reports for most abundantly expressed miRNAs in the ovary of vertebrates (Kang et al., 2013; Wang et al., 2017; Wu et al., 2017; Wang and Ma, 2019). In particular, gga-miR-143-3p, gga-miR-145-5p, gga-miR-26, gga-miR-99, gga-miR-10a, gga-miR-21, gga-miR-148, gga-miR-199, gga-miR-126, gga-miR-125, gga-miR-101 and gga-let-7 family gene clusters were reported as the most abundantly expressed miRNAs in the ovaries of laying chickens (Kang et al., 2013; Wu et al., 2017). In addition, gga-miR-145-5p gene target mRNAs in the ovary of chickens were enriched in TGF-β signaling pathway by regulating the growth and development of primordial follicles (Kang et al., 2013). In mouse, gga-miR-143 homolog was also highly expressed in the ovary and regulates estradiol synthesis (Zhang et al., 2017) thereby controlling ovulation. Loss of gga-miR-145 function in the ovary of mouse model was successfully linked to hyper-activation of primordial follicles, and deregulation of zona pellucida in actively growing follicles (Yang et al., 2013) which might lead to depletion of ovarian reserve. Further, gga-miR-21 in ovaries of chickens was construed to be involved in follicular growth and ovulation (Kang et al., 2013). Taken together, these suggest that miRNAs with abundant expression in the ovary play critical roles in follicular development and ovulation, two processes that regulate egg reproduction in hens.

The main purpose of the present investigation was to identify miRNAs with non-additive expression in the pre-hierarchical ovarian follicles of laying hens. For the first time, our study identified 20 known and novel miRNAs with additive mode of expression, and 50 known and novel miRNAs that demonstrated non-additive expression. Of the 50 DEMiRs that demonstrated non-additive expression, 20 were known while 30 were not previously reported. MicroRNA families that showed non-additive mode of expression include gga-miR-1684, gga-miR-19 and gga-miR-7, which have two members each and gga-miR-34 family which has four members.

MicroRNAs in the gga-miR-1684 family exhibited high-parent dominant expression in both hybrids, while all the four members of the gga-miR-34 family showed low-parent mode of expression. Previous investigation of miRNA transcriptome in the hierarchical follicles of laying chickens revealed that gga-miR-34b and gga-miR-34c members were up-regulated in more efficient laying hens (Wu et al., 2017). In our study, dominance was the major mode of non-additive expression exhibited by known miRNAs (5 high-parent dominant and 14 low-parent dominant). Of note, gga-miR-375 was the only known miRNAs that demonstrated over-dominance mode of expression in the WR hybrid hens. Previously, *in vitro* over-expression study in granulosa cell (GC) line from pig ovary has established that miR-375 regulates E2 synthesis (Yu et al., 2017) and its mode of expression in the present study has confirmed its involvement not only in reproduction but also hybrid vigor for egg laying and clutch traits in hens. However, it is not clear how higher expression of gga-miR-375 in the pre-hierarchical follicles of WR hybrids that demonstrated high heterosis in egg and clutch traits may promote laying of more eggs. Yu et al. has reported co-expression between gga-miR-357 and corticotrophin releasing hormone in the GCs, which synergistically regulate E2 synthesis (Yu et al., 2017). It can be asserted that the higher expression may be exclusive only to the pre-hierarchical follicles. If this was the case, it can be a strategy for preserving follicle pool where only hierarchical follicles will show low expression thereby reducing disrupted follicular selection process typical in low efficient laying birds.

Evidence provided by the result in our study suggests the presence of synergistic effect between miRNAs that exhibited low and high parent dominance in dictating heterosis for egg number and clutch size. Results of the GO and REACTOME enrichment were populated with terms and pathways related to regulation of transcription and gene expression, and suggests the importance of these processes in modulating heterosis for egg number and clutch size in the crossbred laying hens. Further evidences inferred by KEGG enrichment analysis supporting the crucial role of target genes of miRNAs exhibiting non-additive mode of expression are centered in processes related to organization of follicular hierarchy and recruitment. Finely orchestrated follicular development is essential for efficient egg laying typical in commercial hybrid hens. This complex process begins by systematic activation of the quiescent, non-growing primordial follicles to primary oocyte. Follicles that did not follow this cellular fate undergo atresia, thus, reducing follicular reserve. Results of the current study suggests the involvement of cellular senescence in achieving follicle atresia, and genes in the pathway were targeted by miRNAs exhibiting low-parent dominance (gga-miR-19a-3p, gga-miR-1351-5p and gga-miR34c-3p). Hedgehog signaling, the most enriched pathway in the current study, was reported to regulate follicle development and female germ line stem cell proliferation (Jiang et al., 2019). Majority of the target genes enriched in this pathway were targeted by gga-miR-19a-3p and gga-miR-1351-5p, both of which exhibited low-parent dominance in the crossbred hens. Furthermore, pathways for signal transduction including FoxO, WNT, insulin, mTOR, and ErbB were reported to be crucial for follicle activation, growth and development (Kang et al., 2013; Sun et al., 2015; Lee et al., 2016; Tao et al., 2017; Zhao et al., 2018; Zhang et al., 2019; Zhou et al., 2020). Specifically, it was shown that elevated activity of mTORC1 in oocytes causes follicular depletion and premature ovarian failure (Adhikari et al., 2010). Our data corroborates these reports. Genes involved in insulin signaling pathway were reported to be targets for gga-miR-375 (Kang et al., 2013) similar to the observation in the current study.

TGF-β signaling and the genes that enriched the pathway (INHBB, INHBA, PITX2, Activin) support maintenance of ovarian reserve which diminishes owing to either follicle activation, recruitment, maturation and subsequent ovulation, or through atresia (Pelosi et al., 2015). It is asserted here that oocyte maturation for follicles destined to ovulate were facilitated via key pathways including oocyte meiosis, GnRH-signaling and progesterone-mediated oocyte maturation. Many of these pathways were significantly enriched in the ovaries of actively laying geese, and laying chickens with high egg production (Luan et al., 2014; Zhang et al., 2019). Recent investigation reported that focal adhesion and gap junction pathways were enriched in the hypothalamus-pituitary-gonadal tissues, which were linked to high egg production in geese (Wu et al., 2020). Similarly, our data support the involvement of these pathways enriched by genes targeted by miRNAs with non-additive expression in the pre-hierarchical follicles of laying hens.

Based on mRNA-miRNA co-expression network, target genes for gga-miR-19-3p, gga-miR-34-5p, gga-miR-217-5p, gga-miR-135a-5p, gga-miR-205a, gga-miR-375, and gga-miR-7 may underlie heterosis for egg laying in hybrid hens. Specifically, gga-miR-34-5p and gga-miR-205a promote cell apoptosis and were significantly expressed in small yellow follicles of laying chickens and geese (Wu et al., 2020; Hou et al., 2021) while gga-miR-145a-5p promotes premature progesterone release in granulosa cells of pre-hierarchical follicles (McBride et al., 2012). These processes could induce atresia with consequent erosion of follicle reserve. Furthermore, the two candidate miRNAs with high-parent or overdominance expression (gga-miR-375 and gga-miR-7) were associated with regulation of cell proliferation (Yuan et al., 2015), inhibition of synthesis and secretion of gonadotropins (He et al., 2020) essential for finely regulated follicular hierarchy establishment. Taken together, the interplay of miRNAs with non-additive expression and their target genes could underlie heterosis in egg production and clutch traits observed in the hybrid chickens.

## Conclusion

This study was designed to characterize miRNAs with non-additive expression in the follicles of purebred and crossbred hens, and investigate the functions of miRNAs in modulating heterosis for egg number and clutch size. To achieve that aim, mode of miRNA expression was characterized by miRNA sequencing. A total of 50 miRNAs including 30 novel, were found to exhibit non-additive expression. Dominance was the predominant mode of expression exhibited by majority of the miRNAs. Functional analysis of target genes of the known miRNAs with non-additive expression were significantly enriched in GO terms related to regulation of transcription, metabolic processes and gene expression. KEGG pathways enriched in target genes of non-additive genes include hedgehog, cellular senescence, WNT, TGF-beta, progesterone-mediated oocyte maturation, oocyte meiosis, GnRH signaling, signal transduction and transcription regulation, which could be linked to primordial follicle activation, growth and ovulation. mRNA-miRNA co-expression network constructed using mRNA and miRNA suggest gga-miR-19 family, gga-miR-375, gga-miR-205a-3p, gga-miR-375, and gga-miR-7 family are candidate miRNAs that play synergistic roles in maintenance of organized follicular growth and development which may influence heterosis for egg number and clutch size in crossbred hens.

## Materials and methods

### Experimental birds

Experimental procedure for all animal experiments was approved by the Animal Care and Use Committee of the Institute of Animal Science, Chinese Academy of Agricultural Sciences, Beijing (IAS-CAAS). The procedure for the generation of the experimental birds involves crossbreeding of R and W purebred lines to produce offspring of the purebred lines; W, R and their reciprocal crossbred RW and WR. Briefly, semen from R and W purebred sires were artificially inseminated to purebred dams of their lines and the other lines in a reciprocal crossing design to obtain half-sib purebred and crossbred chicks. Four genotypes of chicks were hatched. Female chicks so generated were vaccinated, wing banded and raised. All hens were managed under standard housing and fed appropriate kind of diet specific to age and developmental stage of the birds. The birds were offered unrestricted access to feed and water throughout the period of brooding, rearing and laying.

### Phenotypic data recording

Egg laying was recorded for individual hens once daily. Total egg number and clutch size up to 53 weeks of age were computed for individual hens and selected hens with phenotypic records corresponding to their population average. Least square means for egg number was compared between genetic groups using Tukey-Kramer HSD at *p* < 0.05, using the model below;

$$Y_{ij}=\mu +G_{i}+\xi_{ij}$$

Where $Y_{ij}$ is the phenotype, μ is the population average, $G_{i}$ is the fixed effect of genetic group, and $\varepsilon_{ij}$ is the random error.

Heterosis was calculated using the model below;

$$Heterosis\left(\%\right)=\left[\frac{F_{1}-\left(\frac{P_{1}+P_{2}}{2}\right)}{\frac{P_{1}+P_{1}}{2}}\right]\times 100$$

Where F_1_ is the performance value of the hybrid, P_1_ and P_2_ are the performance values of the two parental lines.

### Tissue collection

Four birds each from the purebred parental and reciprocal crossbred populations (R, W, RW and WR) that have egg laying records corresponding to the average of their populations were exsanguinated by cervical dislocation. After slaughter, the pullets were dissected by ventral midline incision and the intact reproductive tract of the hens were collected, weighed and separated into ovary (and follicles) and oviduct. Pre-hierarchical follicles (4–8 mm in diameter) were sorted and collected from each bird, snap frozen in liquid nitrogen, taken to laboratory and stored at -80 °C until RNA extraction.

### RNA purification and microRNA library preparation

Pre-hierarchical follicles (30–50 mg/sample) were used for total RNA extracted using Trizol reagent (Invitrogen). A total of 16 samples with four each belonging to the W, R, RW and WR were used. RNA concentration and integrity were determined using NanoDrop. ND-1000 (NanoDrop Technologies, Wilmington, DE, United States) and Agilent 2100 Bioanalyzer (Agilent Technologies, CA, United States) respectively. Only RNA sample with concentration >200 ng/μL, RIN >7 and 28S/18S rRNA ratio >1.7 was used for the small RNA library construction. An amount of 1 μg total RNA was used in the construction of each of the libraries (n = 16) using Illumina TruSeq.

### Small RNA sequence data analysis

*In silico* processing of the generated sequence reads was carried out in accordance with established pipelines (Korpelainen et al., 2014; Do et al., 2018). Briefly, raw sequence reads (16 fasta files) were subjected to quality control using FastQC (He et al., 2020) where adapter primers and poor quality reads were removed. Trimming of 3′ and 5′ adapter sequences was achieved using in-house pipeline developed by Annaroad Gene Technology Co., Ltd. (Beijing, China). Only reads with Phred score >20 and sequence read between 18 and 30 nt were retained. Clean reads that passed the quality control criteria were parsed to build a reference genome index. Bowtie 1 (http://bowtie-bio.sourceforge.net/index.shtml) (Langmead et al., 2009) was used to align the built reference genome to GRCg6a (GCA_000002315.5) chicken genome downloaded from the Ensembl database.

### Identification and discovery of known and novel miRNAs

The identification of known miRNAs was performed in miRBase (v22.1) (Andrews, 2017). Discovery of novel miRNAs was achieved after excluding known miRNAs. Reads that mapped to other small RNA species (rRNA, tRNA, snRNA, and snoRNA) Rfam (v.13.0) in the RNA family database (http://rfam.xfam.org/) (Kozomara et al., 2019) and those that mapped to the repeated regions of the genome were excluded. Identification of the repeated regions from the uniquely mapped reads was achieved using RepeatMasker v.4.0.9 (http://repeatmasker.org/cgi-bin/WEBRepeatMasker). MirDeep2 program that implements a probabilistic algorithm based on the miRNA biogenesis model was used to predict novel miRNAs in each library (Kalvari et al., 2018; Friedländer et al., 2012).

### Differential miRNA expression

MiRNAs that met the criteria of being either known or novel were used for differential expression analysis. DEGseq (v1.18.0) package implemented in Bioconductor which follows the assumption of binomial distribution was used for differential gene expression analysis (http://www.bioconductor.org/packages/release/bioc/html/DEGseq.html). Only miRNAs that fulfill the Benjamini and Hochberg criteria for multiple testing correction (false discovery rate (q < 0.05) and |Log_2__ratio| ≥1 were identified as DEMiRs. Differential expression of miRNAs was achieved based on pairwise comparisons of R vs W, R vs RW, R vs WR, W vs RW, and W vs WR. Additionally, the reciprocal crosses were also compared with a synthetic group of mid-parent miRNA expression values, which were calculated by taking the means of normalized gene count from combinations of paternal lines (A = 1/2 (R + W)). Consequently, miRNAs with additive and various forms of non-additive modes of expression were identified. Delineation of DEMiRs into additive, high-parent dominant, low-parent dominant, over-dominant and under-dominant were in accordance with previous studies (Swanson-Wagner et al., 2006; Mai et al., 2019) with little modification. Briefly, additivity occurs when expression of the miRNA was significantly different between the two parental purebred lines (R vs. W Padj.< 0.05, Log_2_FC ≥ 1) and that the miRNA expression in the crossbred was similar to the synthesized mean of their parental purebred lines. The high-parent dominant mode was when expression of the miRNA in the crossbred was significantly higher than one parent but similar to the other parental line. The low-parent dominant mode was when expression in the hybrid was significantly lower than one parental line but similar to the other parental line. Over-dominant mode occurs when miRNA expression in the crossbred was significantly higher than either of the two parental purebred lines. Under-dominance was when gene expression in the hybrid was significantly lower than the two parental purebred lines. This classification was achieved by subjection the DEMiRs to systematic comparisons in Venny 2.0 (Oliveros, 2007).

### Target genes of the DEMiRs prediction and their enrichment

Target genes for known DEMiRs that exhibited non-additive pattern of expression were predicted with TargeScan release 7.2 (http://www.targetscan.org/vert_72/) (Agarwal et al., 2015). Catalogue of such target genes were filtered against the mRNA transcriptome obtained from the pre-hierarchical follicle tissues of the experimental birds. Only mRNAs common in target genes and expressed genes were retained for gene enrichment analysis. The filtered target genes for the non-additively expressed miRNAs in the hybrid chickens were uploaded into g. Profiler (https://biit.cs.ut.ee/gprofiler/gost) (Raudvere et al., 2019) for gene set enrichment of analysis. Gene set enrichment was carried out in Gene Ontology (GO), Kyoto Encyclopedia of Genes and Genomes (KEGG) and Reactome (REAC) databases. The g. profiler algorithm adopts over-representation analysis approach that uses hypergeometric test to measure the significance of functional terms in the input gene list (Raudvere et al., 2019). Significant threshold used was Benjamini–Hochberg corrected FDR threshold at 0.05. Network mapping of interconnectedness of significantly enriched KEGG pathways was achieved using GeneAnalyst (Zhou et al., 2019).

### Construction of mRNA-miRNA co-expression network

We constructed mRNA-miRNA regulatory network in order to identify candidate miRNAs with non-additive expression in the pre-hierarchical follicles of hybrid laying hens. Only mRNAs and miRNAs obtained from RNA sequencing of the samples were used. mRNA-miRNA pairs were delineated if their expression had pairwise correction coefficient of -0.9 or lower. The network was visualized using the cytoscape software.

### Real-time quantitative PCR

The total RNA used for miRNA-sequencing was also used for Real-time quantitative PCR (RT-qPCR) to validate the expression of four non-additively expressed DEMiRs (gga-let-7f-5p, gga-miR-10b-5p, gga-miR-34c-3p and gga-miR-375) following previous report (Agarwal et al., 2015). miScript II RT kit (Qiagen) was used for the RT stage. Approximately 1 μL (1 μg) diluted RNA were added to the reaction mix containing 4 μL 5× miScript RT Buffer, 1 μL miScript Reverse Transcriptase Mix, and 15 μL RNase-free water to a final volume of 20 μL. RT reaction conditions consisted of incubation for 60 min at 37°C, incubating for 5 min at 95°C. Real-time PCR reactions were prepared using miScript SYBR Green PCR based on the manufacturer’s protocol. Briefly, 1 μL of the cDNA was added to the reaction mix containing 10 μL 2 × QuantiTect SYBR Green PCR Master Mix, 2 μL 10× miScript universal Primer, 2 μL 10× miScript Primer Assay to a final volume of 20 μL. The qRT-PCR condition consisted of denaturing at 95 °C for 15 min, followed by 40 cycles consisting 95 °C for 15 s, 55 °C 30 s, 70 °C 30 s. Samples were run in three technical replicates. Relative abundance of the micRNA transcripts was calculated using the 2^-∆∆CT^ method. miRNAs expression result from qRT-PCR and their normalized expression from the TrueSeq were plotted in a histogram after calculating their correlation in each sample.

## Data Availability

The datasets presented in this study can be found in online repositories. The names of the repository/repositories and accession number(s) can be found below: The transcriptome data are available in the Sequence Read Archive (https://www.ncbi.nlm.nih.gov/sra) at NCBI, with the BioProject ID: PRJNA859020 and SRA Accession Number: SAMN29766694-29766709.

## Supplementary material

Supplementary Figure S1 Mapping statistics for the 16 miRNA libraries in parental purebred lines and their reciprocal hybrids.

Supplementary Figure S2 Venn diagram depicting number of DEMiRs with additive mode of expression.

Supplementary Table S1 RNA-seq reads mapped to non-coding regions other than miRNAs expressed in the pre-hierarchical follicles of purebred chicken parental lines (R and W) and their reciprocal hybrids (RW and WR).

Supplementary Table S2 Expressed miRNAs in the pre-hierarchical follicles of purebred chicken parental lines (R and W) and their reciprocal hybrids (RW and WR).

Supplementary Table S3 Mode of expression of novel miRNAs in the pre-hierarchical follicles of purebred chicken parental lines (R and W) and their reciprocal hybrids (RW and WR).

Supplementary Table S4 Target genes for known miRNAs with non-additive mode of expression in the pre-hierarchical follicles of purebred chicken parental lines (R and W) and their reciprocal hybrids (RW and WR).

Supplementary Table S5 GO, KEGG and REAC terms enriched in target genes with non-additive mode of expression in the pre-hierarchical follicles of purebred chicken parental lines (R and W) and their reciprocal hybrids (RW and WR).
