# Supplementary figures and images for: MicroRNAs with non-additive expression in the ovary of hybrid hens target genes enriched in key reproductive pathways that may influence heterosis for egg laying traits
Source: Front Genet. 2022 Sep 30;13:974619. doi: 10.3389/fgene.2022.974619 (PMC9563710; doi:10.3389/fgene.2022.974619)

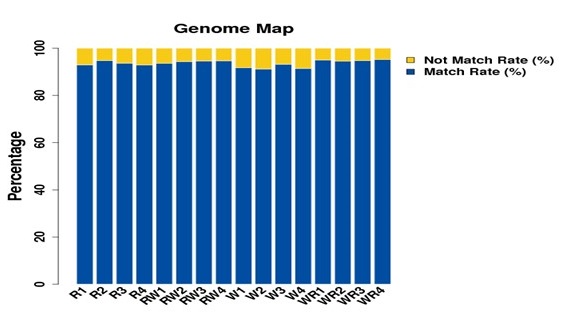

Supplement: Supplementary file 1 [file Image1.JPEG]

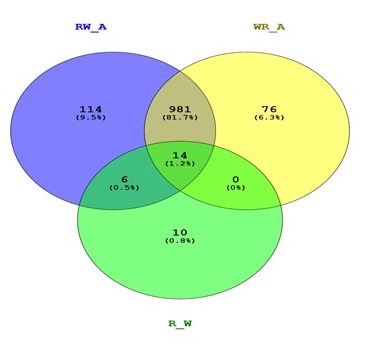

Supplement: Supplementary file 2 [file Image2.JPEG]
